# Therapeutic Decision Making in Acute Stroke due to Carotid Artery Dissection: A Potential Role for Percutaneous Vascular Intervention following Intravenous Thrombolysis

**DOI:** 10.1155/2013/121696

**Published:** 2013-02-21

**Authors:** J. B. Lewis, Á. Merwick, R. Ó. Laoide, A. O'Hare, C. McGuigan

**Affiliations:** ^1^Department of Neurology, St. Vincent's University Hospital, Elm Park, Dublin, Ireland; ^2^Department of Neurology, Beaumont Hospital, Co. Dublin, Ireland; ^3^Department of Radiology, St. Vincent's University Hospital, Elm Park, Dublin, Ireland; ^4^Department of Neuroradiology, Beaumont Hospital, Co. Dublin, Ireland

## Abstract

Internal carotid artery dissection (ICAD) is an important cause of acute ischemic stroke in younger patients. Potential acute treatments include anticoagulation, intravenous thrombolysis (IVT), and endovascular thrombectomy (ET). We report a case where the use of IVT followed by ET resulted in a good clinical outcome in a patient with tandem internal carotid and middle cerebral artery occlusion following ICAD.

## 1. Introduction

A 31-year-old right-handed woman, who was 4-month postpartum, presented to the emergency department with a thirty-five-minute history of left-sided face, arm and leg weakness, along with slurred speech. She reported right-sided neck pain for the preceding three days, with no history of trauma or neck manipulation. Two days prior to presentation, she had experienced transient loss of vision in her right eye.

On examination, she had left upper motor neuron pattern facial weakness, left upper and lower limb MRC grade 0/5 power, sensory inattention, and cumulative NIH Stroke Scale (NIHSS) score of 14. Horner's syndrome was absent.

**Figure 1:**
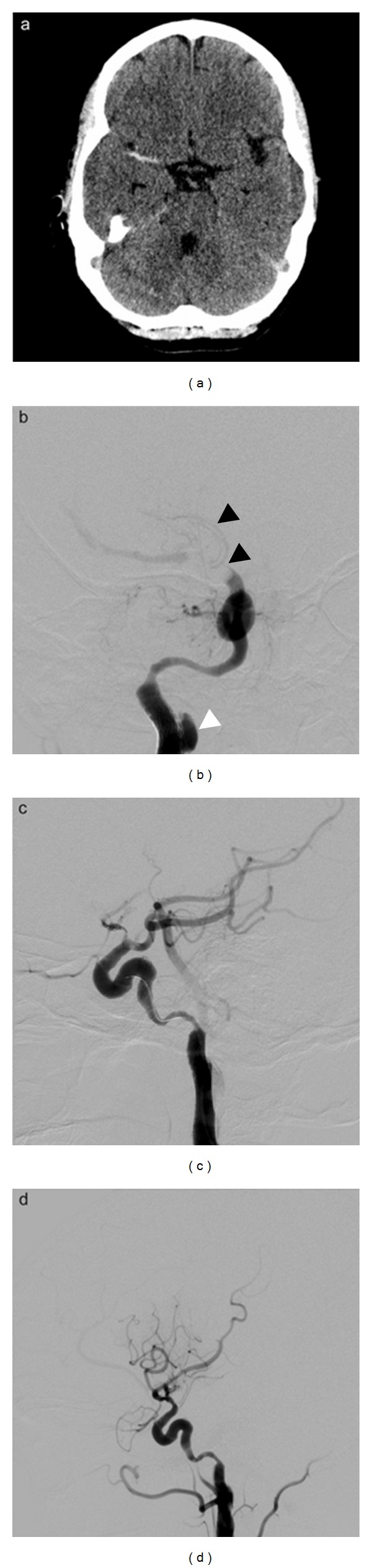
(a) Prethrombolysis CT showing hyperdense vessel sign in right MCA. (b) Angiogram after right ICA stenting and before Solitaire AB deployment showing pseudoaneurysm at the dissection site (white arrowhead) and thrombus within the ACA and M1 (black arrowheads). (c) Solitaire AB deployment with stent in situ. (d) Angiogram after clot retrieval from right MCA showing recanalization and reperfusion.

CT brain imaging performed 1 hour after symptom onset showed a hyperdense middle cerebral artery (MCA) sign (Figure 1(a)), and CT angiogram showed a right internal carotid artery dissection (ICAD). MRI confirmed the internal carotid artery dissection and demonstrated restricted diffusion limited to the lenticulostriate distribution of the MCA.

The intravenous thrombolysis (IVT) was initiated 2 hours 20 minutes following symptom onset, but following IV tPA administration the patient continued to display persistent left hemiparesis, dysarthria, and sensory inattention. NIHSS score was 10.

The patient was transferred to a hospital with interventional neuroradiology expertise in view of the persisting disability and presumed persistent proximal artery occlusion. Informed consent was obtained and the patient was placed under general anaesthesia. The initial angiogram via a 5 Fr catheter showed a normal left carotid, left anterior circulation, vertebral artery, and posterior circulation. Absence of cross-flow via the anterior communicating artery precluded the assessment of the right anterior circulation from the left side. There was some retrograde collateral filling of the right anterior cerebral and MCA territories.

At 6 hours 12 minutes following symptom onset, the right common carotid artery was accessed via an 8 Fr flow arresting guide catheter. Initial angiography demonstrated tapered occlusion of the proximal right internal carotid artery (ICA) consistent with dissection. A microwire was successfully passed beyond the dissection after several attempts. After confirming the intraluminal position of the wire, a 6 mm × 30 mm carotid stent was placed across the occlusion. Repeat angiography revealed a patent right ICA with a pseudoaneurysm at the dissection site and thrombus in the right anterior cerebral artery (ACA) and M1 (Figure 1(b)). Three passes were made with a 4 mm × 20 mm Solitaire AB (ev3 Inc, Irvine, C.A, USA), a self-expanding and fully retrievable stent and ACA and M1 thrombus retrieved (Figure 1(c)). Early neurological improvement was noted after procedure. At discharge 7 days later the patient's NIHSS score was 2, and she made an excellent functional recovery.

## 2. Discussion

Acute treatment of stroke due to arterial dissection and occlusion is a challenge in clinical practice. Intravenous thrombolysis has been shown to improve long-term outcome in stroke patients, but patients with carotid artery dissection (CAD) receiving IVT have poorer outcomes than those without CAD [1–3]. Treatment guidelines do not advise against IVT in proximal artery occlusion (PAO) with ICA dissection, but PAO is an independent predictor of poor outcome after IVT [4, 5]. Patients with PAO have worse functional outcome and higher mortality compared to those with normal CT angiogram [4, 5]. However, it does not appear that ICAD is an additional risk factor for intracranial bleed or stroke recurrence in the context of IVT therapy, and secondary complications due to extension of wall hematoma have not been observed in a number of studies [3, 6, 7]. 

In patients where the response to IVT has been poor or incomplete, endovascular thrombectomy (ET) may lead to a more favourable long-term outcome compared to pharmacological therapy alone, though there is a scarcity of randomised trial or registry data available [8, 9]. Despite this paucity of data, stent placement and other ET techniques have been shown to be effective in patients where medical therapy has failed [10, 11], with good long-term outcomes in both traumatic and spontaneous dissections [12]. In severe or complete ICA occlusion with tandem MCA infarction, ET was demonstrated to improve the patient outcome as compared to medical therapy alone [13]. 

When the clinician is faced with treating acute stroke due to arterial dissection and occlusion, consideration of all therapeutic options may help prevent serious long-term disability and lead to a more favourable long-term-result. Therefore, randomized trials of ET are needed to establish its efficacy and safety as well as guidance regarding optimal time window for its potential use.
